# Fast fluoride ion conduction of NH_4_(Mg_1-*x*_Li_*x*_)F_3-*x*_ and (NH_4_)_2_(Mg_1-*x*_Li_*x*_)F_4-*x*_ assisted by molecular cations

**DOI:** 10.1038/s41598-022-09835-0

**Published:** 2022-04-08

**Authors:** Kota Motohashi, Yosuke Matsukawa, Takashi Nakamura, Yuta Kimura, Naoaki Kuwata, Yoshiharu Uchimoto, Koji Amezawa

**Affiliations:** 1 Graduate School of Engineering, Tohoku University, 6-6 Aramaki Aza Aoba, Aoba-ku, Sendai, Miyagi 980-8579 Japan; 2 Institute of Multidisciplinary Research for Advanced Materials, Tohoku University, 2-1-1 Katahira Aoba-ku, Sendai, Miyagi 980-8577 Japan; 3 National Institute for Materials Science, 1-1 Namiki, Tsukuba, Ibaraki 305-0044 Japan; 4 Graduate School of Human and Environmental Studies, Kyoto University, Yoshida-nihonmatsu-cho, Sakyo-ku, Kyoto, 606-8501 Japan; 5 Present Address: Graduate School of Engineering, Osaka Prefecture University, 1-1 Gakuen-cho, Naka-ku, Sakai, Osaka 599-8531 Japan

**Keywords:** Solid-state chemistry, Batteries

## Abstract

Aiming development of the fast anion conductors, we proposed a new material design using flexible molecular cation as a host cation, and demonstrated it with fluoride ion conduction in NH_4_MgF_3_ and (NH_4_)_2_MgF_4_ based materials. Dominant fluoride ion conduction with relatively high conductivities of 4.8 × 10^–5^ S cm^−1^ and 8.4 × 10^–6^ S cm^−1^ were achieved at 323 K in (NH_4_)_2_(Mg_0.85_Li_0.15_)F_3.85_ and NH_4_(Mg_0.9_Li_0.1_)F_2.9_, respectively. It is implied that the molecular cation in the host lattice can assist the anion conduction. Our findings suggest molecular cation-containing compounds can be attractive material groups for fast anion conductors.

## Introduction

Developing high energy density batteries are an urgent issue for establishing environmentally-friendly and sustainable society. All-solid-state fluoride ion batteries (ASSFIBs) are one of promising batteries because of their potential of high energy density^1–4^. The energy density of ASSFIBs is theoretically expected to reach 5000 Wh L^−1^. However, state-of-the-art ASSFIBs still have many problems, for instance, the gap between theoretical and practical discharge/charge capacities, the poor cycling performance, the high operating temperature, the insufficient operating voltage, and so on^5,6^. One major reason for such poor performances of the present ASSFIBs is the lack of suitable solid electrolytes having high ionic conductivity and thermochemical stability. PbSnF_4_ shows the highest ionic conductivity, 1.6 × 10^–3^ S cm^−1^ at room temperature, among already-known solid-state fluoride ion conductors. However, this material is unstable under the high operating voltage due to the narrow potential window.

There are several strategies for development of solid electrolytes. One is the use of highly disordered structure advantageous for high ionic conduction. Another is the introduction of the mobile ionic defects (such as vacancies or interstitial ions) by doping aliovalent ions. Among fluoride ion conductors, PbSnF_4_, RbSnF_3_, and β-PbF_2_ are the materials developed based on the former strategy^7–10^. On the other hand, the tysonite-type La_1-*x*_Ba_*x*_F_3-*x*_ and Sm_1-*x*_Ca_*x*_F_3-*x*_ and the fluorite-type Sn_1-*x*_K_*x*_F_2-*x*_ and Ba_1-*x*_La_*x*_F_2+*x*_ are the materials based on the latter one^11–14^. Although various fluoride ion conductors are previously reported^15,16^, further material explorations for sufficiently high fluoride ion conductivity are required to realize ASSFIBs.

Excellent cation conduction has been reported in some materials containing molecular anions such as PO_4_^3−^, SiO_4_^4−^, PS_4_^3−^, and etc. proton conductors like CsH_2_PO_4_ and lithium ion conductors like Li_3_PO_4_-Li_4_SiO_4_ and Li_3_PS_4_ are the typical examples^17–20^. High cation conductivity in these materials is considered to be caused by unique size, structure, and dynamics of molecular ions, resulting in extension of the bottleneck for ion conduction, reduction of the interaction between the host and carrier ions, and assistance of the ion conduction by the rotation of the molecular ions^21^, and so on. Considering these, a similar strategy can be applied to develop novel anion conductors. There had been some attempts to develop new fluoride ion conductors containing molecular ions such as NH_4_^+^ in NH_4_SnF_3_^22^. However, the role of molecular cations for anion conduction has not been well examined. It is therefore interesting to systematically investigate the potential of materials containing molecular cations as fast anion conductors.

In this study, perovskite and layered perovskite fluorides containing NH_4_^+^ as a molecular cation, NH_4_MgF_3_ and (NH_4_)_2_MgF_4_, are selected as targets of materials^23^. NH_4_(Mg_1-*x*_Li_*x*_)F_3-*x*_ and (NH_4_)_2_(Mg_1-*x*_Li_*x*_)F_4-*x*_ were prepared with the intention of introducing fluoride ion vacancies by the substitution of Li^+^ for Mg^2+^, and their electrical conduction properties were studied. In comparison, the conductivities of perovskite and layered perovskite containing K^+^ as the A-site cation, K(Mg_0.9_Li_0.1_)F_2.9_ and K_2_(Mg_0.9_Li_0.1_)F_3.9_, were examined. Since the ionic radius of K^+^ (1.64 Å) is similar to the effective radius of NH_4_^+^ (1.46 Å)^24^, the influence of the molecular ions on the ionic conductivity can be discussed.

NH_4_(Mg_1-*x*_Li_*x*_)F_3-*x*_ and (NH_4_)_2_(Mg_1-*x*_Li_*x*_)F_4-*x*_ were synthesized by solid state reaction methods. The obtained powders and pressed samples were characterized by X-ray diffraction (XRD), scanning electron microscopy (SEM) observation, electron probe micro analyzer (EPMA), and nuclear magnetic resonance (NMR) spectroscopy. The thermal stabilities of NH_4_MgF_3_ and (NH_4_)_2_MgF_4_ were examined by thermogravimetry (TG). The electrical conductivities of the pellets were measured by AC electrochemical impedance spectroscopy (EIS). To confirm the dominant fluoride ion conduction, AC EIS and DC polarization measurements were performed with the fluoride ion conducting cell. In order to cross-check the dominant fluoride ion conduction, electromotive force (*emf*) measurements of the fluorine concentration cell, M_1_F_*x*_-M_1_/sample/M_2_F_*x*’_-M_2_ (M_1, 2_: metal, M_1, 2_F_*x*, *x*’_: metal fluoride), were performed. Details are given in the supplementary information.

## Results

**Figure 1:**
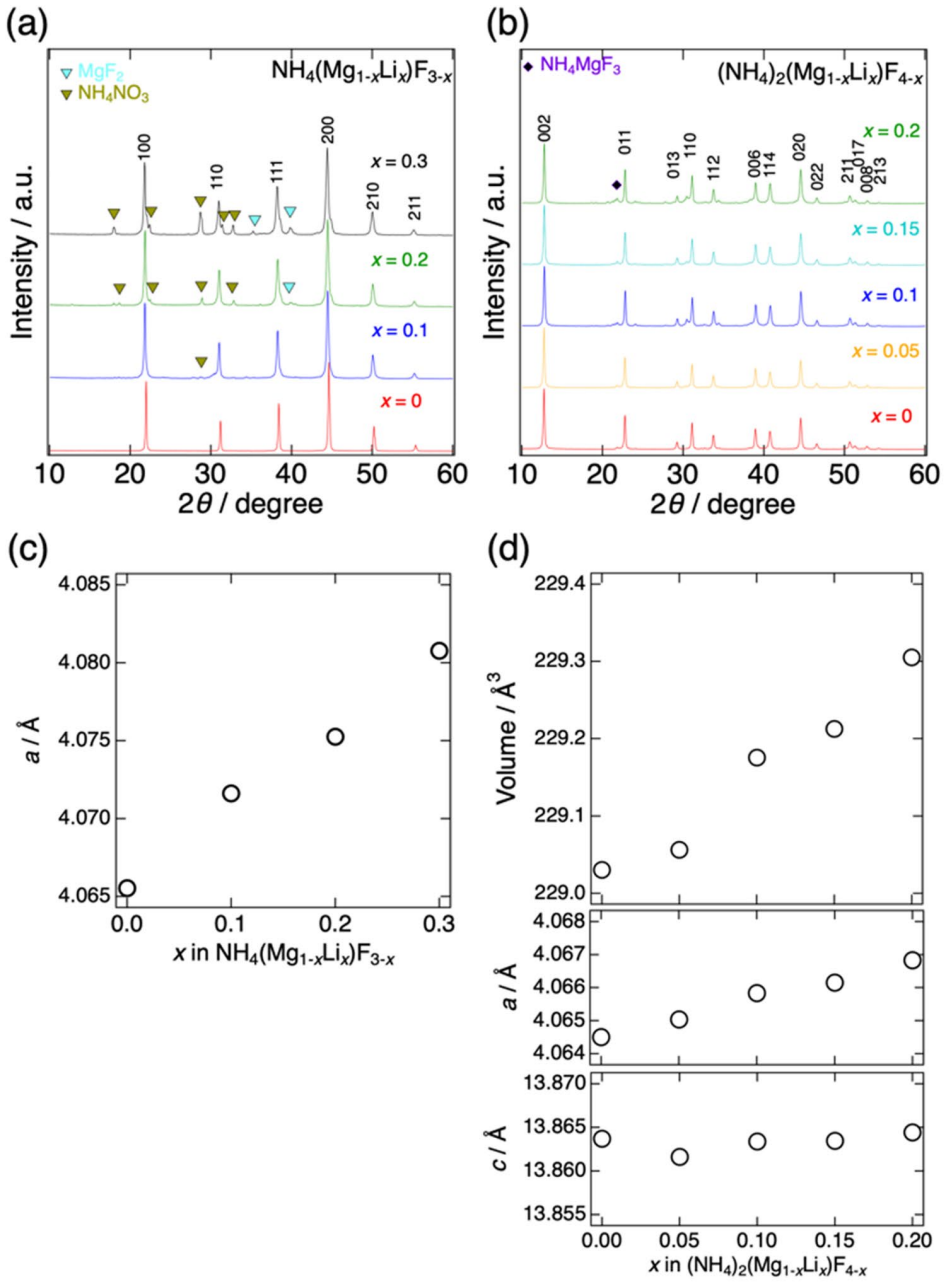
X-ray diffraction patterns of (**a**) NH_4_(Mg_1-*x*_Li_*x*_)F_3-*x*_ (*x* = 0, 0.1, 0.2, and 0.3) and (**b**) (NH_4_)_2_(Mg_1-*x*_Li_*x*_)F_4-*x*_ (*x* = 0, 0.05, 0.1, 0.15, and 0.2). Relation between the Li content and lattice parameters of (**c**) NH_4_(Mg_1-*x*_Li_*x*_)F_3-*x*_ and (**d**) (NH_4_)_2_(Mg_1-*x*_Li_*x*_)F_4-*x*_. The lattice parameters were calculated assuming *Pm* $\overline{3 }$ *m* structure for NH_4_(Mg_1-*x*_Li_*x*_)F_3-*x*_ and *I*4/*mmm* structure for (NH_4_)_2_(Mg_1-*x*_Li_*x*_)F_4-*x*_.

Figure 1a,b show the XRD patterns of (a) NH_4_(Mg_1-*x*_Li_*x*_)F_3-*x*_ and (b) (NH_4_)_2_(Mg_1-*x*_Li_*x*_)F_4-*x*_. The most of XRD peaks could be indexed with the cubic (*Pm* $\overline{3 }$ *m*) symmetry for NH_4_(Mg_1-*x*_Li_*x*_)F_3-*x*_ and the tetragonal symmetry (*I*4/*mmm*) for (NH_4_)_2_(Mg_1-*x*_Li_*x*_)F_4-*x*_. In NH_4_(Mg_1-*x*_Li_*x*_)F_3-*x*_, the diffraction peaks of the cubic phase gradually shifted to lower angle with increasing the Li content. This indicated that larger Li^+^ (0.76 Å) was substituted into the smaller Mg^2+^ (0.72 Å) sites. The lattice parameters of NH_4_(Mg_1-*x*_Li_*x*_)F_3-*x*_ and (NH_4_)_2_(Mg_1-*x*_Li_*x*_)F_4-*x*_ were calculated from the diffraction angles and were plotted in Fig. 1c,d as a function of the Li content. Except for the *c*-axis of (NH_4_)_2_(Mg_1-*x*_Li_*x*_)F_4-*x*_, the lattice parameters changed monotonically with the Li content in NH_4_(Mg_1-*x*_Li_*x*_)F_3-*x*_ and (NH_4_)_2_(Mg_1-*x*_Li_*x*_)F_4-*x*_ phases, suggesting that solid solution is formed at least within the compositional range of 0 < *x* < 0.3 in NH_4_(Mg_1-*x*_Li_*x*_)F_3-*x*_ and 0 < *x* < 0.2 in (NH_4_)_2_(Mg_1-*x*_Li_*x*_)F_4-*x*_ and the solubility limit of Li is higher than 30 mol% in NH_4_(Mg_1-*x*_Li_*x*_)F_3-*x*_ and 20 mol% in (NH_4_)_2_(Mg_1-*x*_Li_*x*_)F_4-*x*_. Small diffraction peaks of NH_4_NO_3_ could be found in some compositions, especially in NH_4_(Mg_0.8_Li_0.2_)F_2.8_. In order to investigate the state and location of the impurity, the SEM observation and EPMA analysis were carried out. The results for NH_4_(Mg_0.8_Li_0.2_)F_2.8_ were presented in Figs. S1. The impurity, possibly NH_4_NO_3_, was observed as indicated by the yellow circles in Fig. S1. However, since the impurity particles seemed to exist sparsely from the main compound and their amount was not significant, the influences of the impurity on the observed ionic conductivities were supposed as negligibly small.

The SEM images of the cross sections of the pressed samples of NH_4_(Mg_0.8_Li_0.2_)F_2.8_ and (NH_4_)_2_(Mg_0.85_Li_0.15_)F_3.85_ were shown in Fig. S2. The pellets seemed dense as just pressed, and the relative densities of all the pellets were approximately 75%.

Figure S3 (a) and (b) show the results of TG measurement. NH_4_MgF_3_ and (NH_4_)_2_(Mg_0.8_Li_0.2_)F_3.8_ were stable below approximately 443 and 413 K, respectively. As shown in Figs. S3 (c) and (d), XRD analysis indicated that NH_4_MgF_3_ was decomposed to MgF_2_ at around 443 K and (NH_4_)_2_MgF_4_ was decomposed into NH_4_MgF_3_ and MgF_2_ near 413 K forming NH_4_F gas.

**Figure 2:**
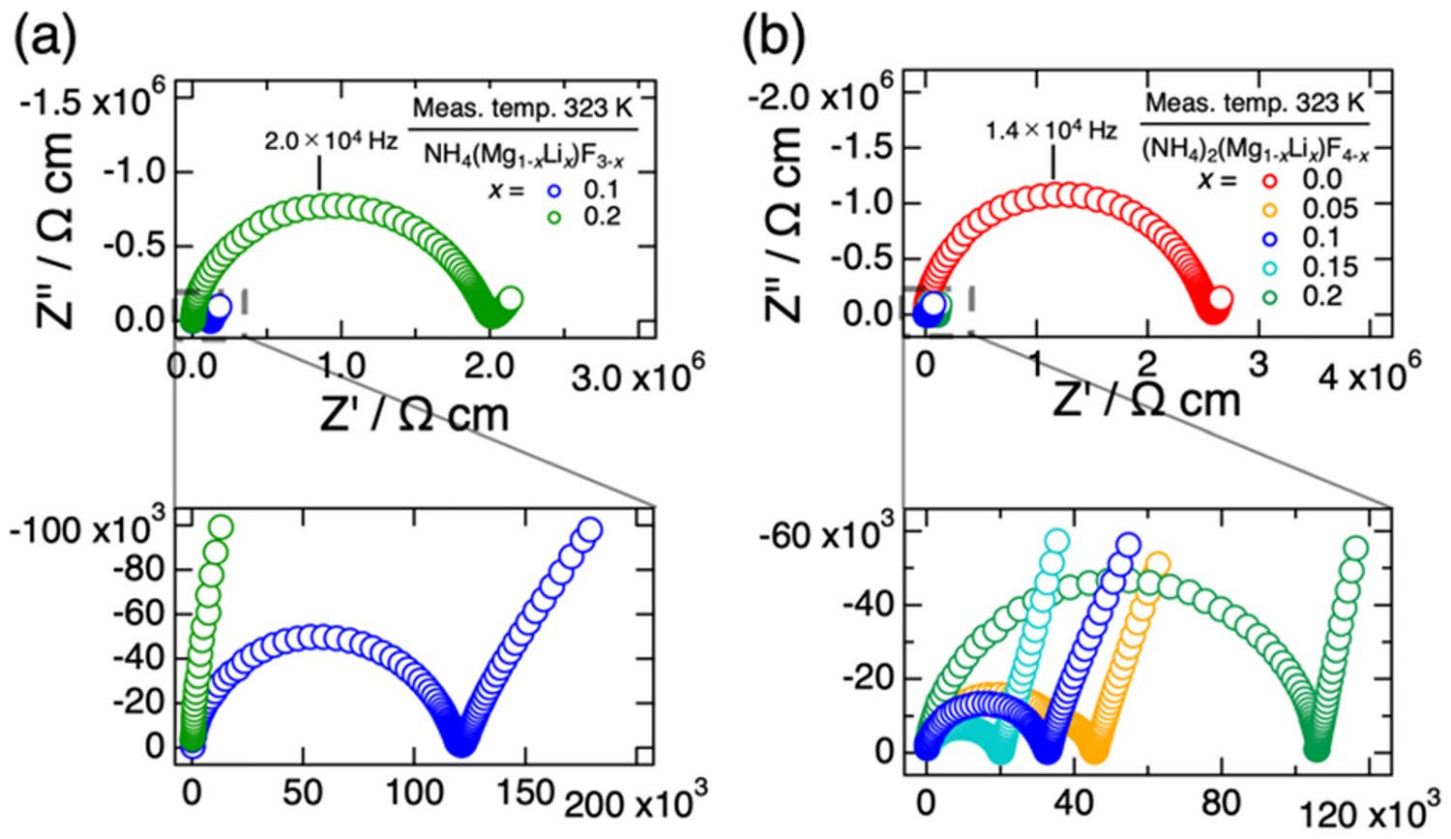
Nyquist plots of (**a**) NH_4_(Mg_1-*x*_Li_*x*_)F_3-*x*_ (*x* = 0.1 and 0.2) and (**b**) (NH_4_)_2_(Mg_1-*x*_Li_*x*_)F_4-*x*_ (*x* = 0, 0.05, 0.1, 0.15, and 0.2) measured at 323 K in N_2_ gas.

**Figure 3:**
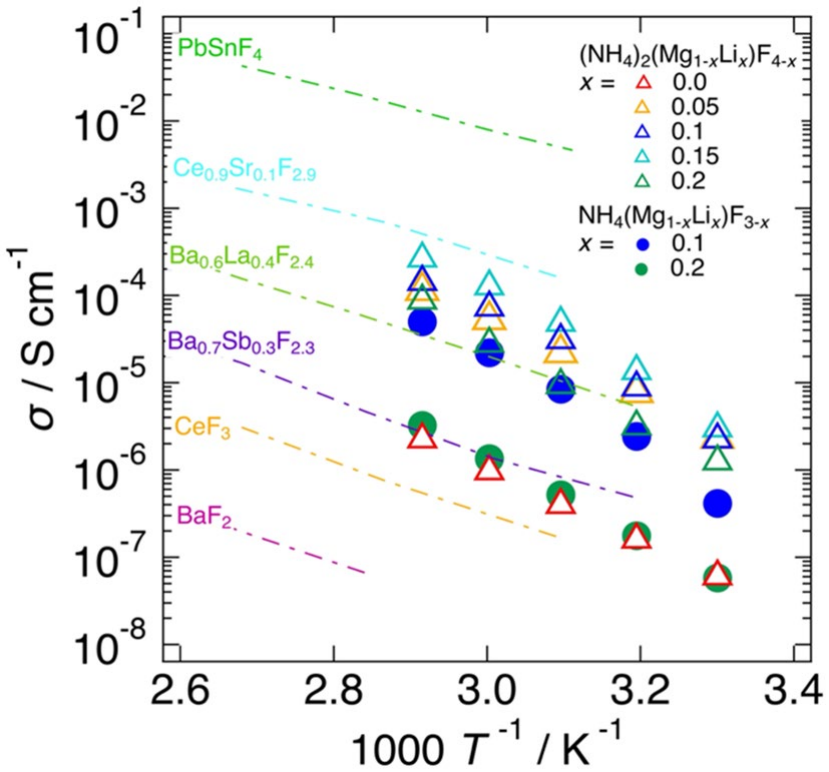
Temperature dependence of the electrical conductivities of NH_4_(Mg_1-*x*_Li_*x*_)F_3-*x*_ (*x* = 0.1 and 0.2) and (NH_4_)_2_(Mg_1-*x*_Li_*x*_)F_4-*x*_ (*x* = 0, 0.05, 0.1, 0.15, and 0.2). Reported electrical conductivities of typical fluoride ion conductors are also plotted in comparison^3,27–29^.

Figure 2 show Nyquist plots observed with (a) NH_4_(Mg_1-*x*_Li_*x*_)F_3-*x*_ and (b) (NH_4_)_2_(Mg_1-*x*_Li_*x*_)F_4-*x*_ at 323 K. Although the results are not given in Fig. 2, only scattered signals were observed in EIS measurements with non-doped NH_4_MgF_3_, indicating its extremely low electrical conductivity. On the other hand, the Li-doped samples showed typical impedance responses of an ionic conductor with blocking electrodes, *e.g.* a semicircle in the high frequency region and a sharp spike in the low frequency region. These impedance behaviours suggested ionic conductivity in these samples. The total resistance of the sample including the bulk and grain boundary resistances was determined from the semicircle in high frequency region. Figure 3 shows temperature dependences of the electrical conductivities of NH_4_(Mg_1-*x*_Li_*x*_)F_3-*x*_ and (NH_4_)_2_(Mg_1-*x*_Li_*x*_)F_4-*x*_. The conductivities were enhanced by Li-doping, but they showed the maximum and decreased with further increasing the Li content. At 323 K, the maximum conductivity was observed at *x* = 0.1 for NH_4_(Mg_1-*x*_Li_*x*_)F_3-*x*_ (8.4 × 10^–6^ S cm^−1^) and at *x* = 0.15 for (NH_4_)_2_(Mg_1-*x*_Li_*x*_)F_4-*x*_ (4.8 × 10^–5^ S cm^−1^). The decrease in electrical conductivity in highly doped samples is considered to be caused by cluster formation or ordering of fluoride ions and vacancies, and etc.^25,26^. The fact that the conductivities showed the maximum at a certain Li concentration also indicated that the presence of the impurities did not affect the conductivity enhancement of NH_4_(Mg_1-*x*_Li_*x*_)F_3-*x*_ and (NH_4_)_2_(Mg_1-*x*_Li_*x*_)F_4-*x*_, because the amount of the impurities monotonically increased with increasing the Li concentration.

**Figure 4:**
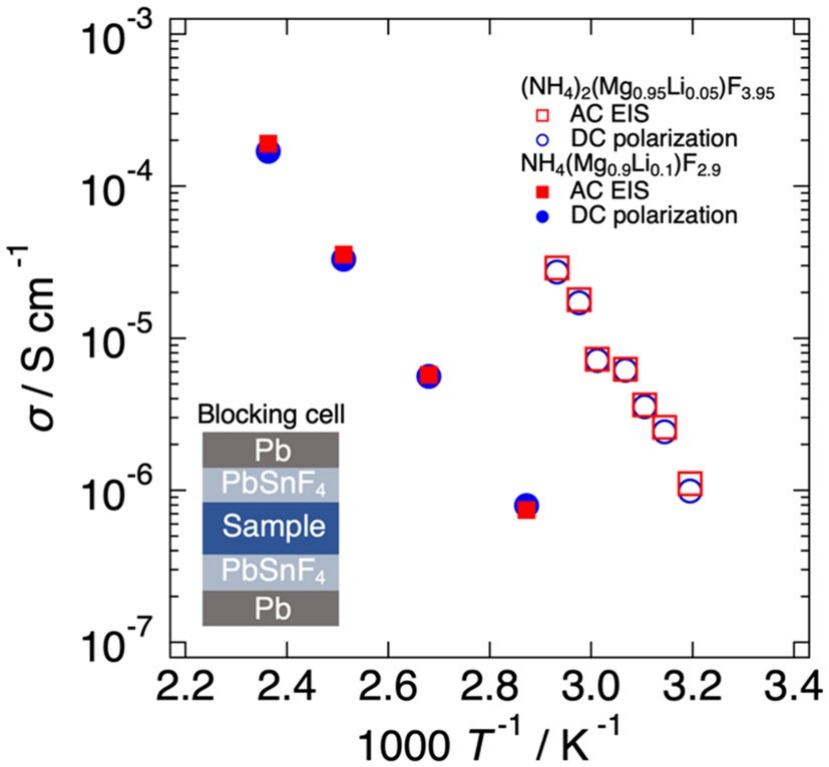
Temperature dependence of conductivities of NH_4_(Mg_0.9_Li_0.1_)F_2.9_ and (NH_4_)_2_(Mg_0.95_Li_0.05_)F_3.95_ measured by AC electrochemical impedance spectroscopy and DC polarization methods with a Pb/PbSnF_4_/sample/PbSnF_4_/Pb cell.

In order to confirm dominant fluoride ion conduction in the investigated materials, we prepared a blocking cell consisting of Pb/PbSnF_4_/sample/PbSnF_4_/Pb. Since PbSnF_4_ is an almost pure fluoride ion conductor, this cell conducts only fluoride ion under steady-state DC bias, while the AC conductivity of the cell includes the contribution of all mobile carriers in the sample. Thus, if the conductivities measured by AC EIS and DC polarization methods are comparable, it can be concluded the dominant carrier is fluoride ion. The voltage transient curves observed in DC polarization measurements with a Pb/PbSnF_4_/samples/PbSnF_4_/Pb at various temperatures are shown in Figs. S4b–e and S5b–h. The measured voltages were considerably increased immediately after the DC polarization and then gradually increased with time. From the impedance spectra shown in Figs. S4(a) and S5(a), the relaxation times for electrical conduction in NH_4_(Mg_0.9_Li_0.1_)F_2.9_ and (NH_4_)_2_(Mg_0.95_Li_0.05_)F_3.95_ were faster than 10^–1^ s. Thus, the gradual increase of the voltage might be mainly caused by the formation of resistive interphases by the decomposition of PbSnF_4_ at the PbSnF_4_/current-corrector interface. Therefore, the DC conductivity of the blocking cell was evaluated from the current and the voltage drop observed at 1 s after applying DC current. Figure 4 shows temperature dependence of conductivities of NH_4_(Mg_0.9_Li_0.1_)F_2.9_ and (NH_4_)_2_(Mg_0.95_Li_0.05_)F_3.95_ measured by AC EIS and DC polarization methods with a Pb/PbSnF_4_/sample/PbSnF_4_/Pb cell. The conductivities by AC EIS and DC polarization methods were comparable both NH_4_(Mg_0.9_Li_0.1_)F_2.9_ and (NH_4_)_2_(Mg_0.95_Li_0.05_)F_3.95_. Thus, it can be concluded that the dominant carrier was fluoride ion both in NH_4_(Mg_1-*x*_Li_*x*_)F_3-*x*_ and (NH_4_)_2_(Mg_1-*x*_Li_*x*_)F_4-*x*_.

In Fig. 3, the conductivities of conventional fluoride ion conductors are shown by dash-dotted lines^3,27–29^. The fluorides investigated in this work exhibited relatively high ionic conductivity, although not as high as that of the best fluoride ion conductor, PbSnF_4_. It is also noteworthy that pressed samples of NH_4_(Mg_1-*x*_Li_*x*_)F_3-*x*_ and (NH_4_)_2_(Mg_1-*x*_Li_*x*_)F_4-*x*_ showed relatively high conductivities without sintering. This can be a great advantage for the fabrication of all-solid-state batteries. The activation energies of NH_4_(Mg_1-*x*_Li_*x*_)F_3-*x*_ and (NH_4_)_2_(Mg_1-*x*_Li_*x*_)F_4-*x*_ were approximately 1.0 eV, as summarized in Table S1. The activation energies of NH_4_(Mg_1-*x*_Li_*x*_)F_3-*x*_ and (NH_4_)_2_(Mg_1-*x*_Li_*x*_)F_4-*x*_ were approximately 0.3–0.4 eV higher than those of the reported typical fluoride ion conductors.

In the case of the layered perovskite structure, interstitial anions sometimes can be mobile, as interstitial oxygens in Ln_2_NiO_4+*d*_ (Ln = rare earth)^30^. Based on this idea, the introduction of interstitial fluoride ions was tried for the layered perovskite (NH_4_)_2_MgF_4_ by partially substituting trivalent cation Sc^3+^ for Mg^2+^. However, as shown in Fig. S6, this trial was not effective for improving the ionic conductivity of (NH_4_)_2_MgF_4_.

**Figure 5:**
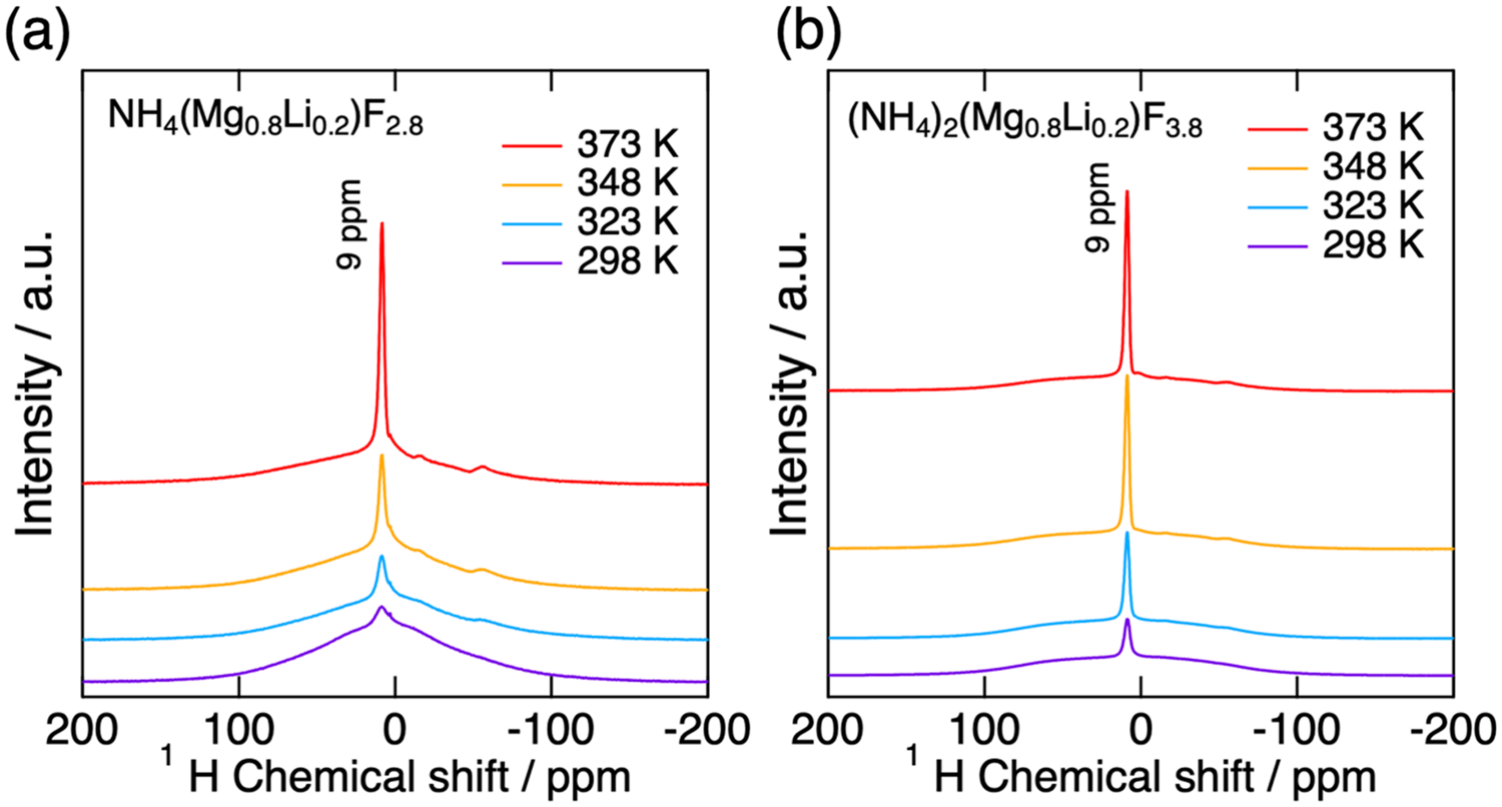
^1^H NMR spectra of (**a**) NH_4_(Mg_0.8_Li_0.2_)F_2.8_ and (**b**) (NH_4_)_2_(Mg_0.8_Li_0.2_)F_3.8_ at various temperatures.

In order to demonstrate the influence of the molecular cations on the anionic conductivity, K(Mg_0.9_Li_0.1_)F_2.9_ having the same crystal structures was prepared. The lattice constant of K(Mg_0.9_Li_0.1_)F_2.9_ was 3.989 Å which was comparable with NH_4_(Mg_0.9_Li_0.1_)F_2.9_, 4.072 Å. The electrical conductivities of K(Mg_0.9_Li_0.1_)F_2.9_ and K_2_(Mg_0.9_Li_0.1_)F_3.9_ were considerably low, 5.2 × 10^–6^ S cm^−1^ at 789 K and 7.3 × 10^–5^ S cm^−1^ at 717 K, respectively (Fig. S7). This demonstrated that NH_4_^+^ in the host lattice can assist the fluoride ion conduction. At this moment, the reason for the conductivity enhancement by the substitution of K^+^ for NH^4+^ is not clear. One likely hypothesis is that the rotational motion of NH_4_^+^ assists the fluoride ion conduction. Figure 5 presents ^1^H NMR spectra of NH_4_(Mg_0.8_Li_0.2_)F_2.8_ and (NH_4_)_2_(Mg_0.8_Li_0.2_)F_3.8_ at various temperatures. A peak was observed at 9 ppm for both NH_4_(Mg_0.8_Li_0.2_)F_2.8_ and (NH_4_)_2_(Mg_0.8_Li_0.2_)F_3.8_. This peak gradually narrowed as temperature increased. As already discussed, the dominant charge carrier in both of NH_4_(Mg_0.8_Li_0.2_)F_2.8_ and (NH_4_)_2_(Mg_0.8_Li_0.2_)F_3.8_ is confirmed to be fluoride ion, meaning the conduction of NH_4_^+^ or proton is negligible. Thus, the narrowing of the ^1^H NMR peak seen in Fig. 5 is considered due to the rotational or reorientational motions of NH_4_^+^. Actually, in (NH_4_)_2_MgF_4_, the rotational motion of NH_4_^+^ was suggested in literature^31^. The rotation of NH_4_^+^ can induce extension of the bottleneck for anion conduction, reduction of the interaction between the host and carrier ions, or assistance of anion hopping, as happened in cation conductors containing molecular anions^17,32^. Such influences by the molecular cation might enhance the fluoride ion conduction in NH_4_MgF_3_ and (NH_4_)_2_MgF_4_ based materials.

In this work, we succeeded to achieve relatively high fluoride ion conductivity in compounds containing molecular cations, NH_4_(Mg_1-*x*_Li_*x*_)F_3-*x*_ and (NH_4_)_2_(Mg_1-*x*_Li_*x*_)F_4-*x*_, by introducing fluoride ion vacancies. It was suggested that the molecular cation in the host lattice might assist anion conduction. The findings of this works suggested that compounds containing molecular cations can be new host materials for fast anion conductors.

## Conclusion

NH_4_(Mg_1-*x*_Li_*x*_)F_3-*x*_ and (NH_4_)_2_(Mg_1-*x*_Li_*x*_)F_4-*x*_ were found to exhibit relatively high fluoride ion conductivities of 8.4 × 10^–6^ (*x* = 0.1) and 4.8 × 10^–5^ (*x* = 0.15) S cm^−1^ at 323 K, respectively. The major conduction carrier was identified as fluoride ion. This work demonstrated that compounds containing molecular cations, like hybrid organic–inorganic perovskites, can be a promising material group for noble anion-conducting materials.

## Methods

### Synthesis and characterization

NH_4_(Mg_1-*x*_Li_*x*_)F_3-*x*_ and (NH_4_)_2_(Mg_1-*x*_Li_*x*_)F_4-*x*_ were synthesized from 3MgCO_3_•3H_2_O (99.9%, Kojundo Chemical Laboratory Co., LTD., Japan), NH_4_F (97.0 + %, Wako Pure Chemical Industries, Ltd., Japan) and LiNO_3_ (99.9%, Wako Pure Chemical Industries, Ltd., Japan) by a solid state reaction. For the synthesis of the compounds, excess amount of NH_4_F was required to compensate the evaporation of NH_4_F during the calcination. Figure S8 shows the products obtained with different molar ratios of NH_4_MgF_3_. When the mixing ratio was 1:7, the single phase of the perovskite NH_4_MgF_3_ was obtained, while impurities including MgF_2_ were observed with the mixing ratios below 1 : 6, suggesting the lack of NH_4_^+^. Considering these results, raw material powders were mixed with a molar ratio of Mg : Li : F = (1-*x*) : *x* : 7. The mixture was calcined at 453 K for NH_4_(Mg_1-*x*_Li_*x*_)F_3-*x*_ and 433 K for (NH_4_)_2_(Mg_1-*x*_Li_*x*_)F_4-*x*_ for 2—8 h under Ar gas flow. In order to remove remaining NH_4_F, the mixtures were additionally calcined at 433 K for NH_4_(Mg_1-*x*_Li_*x*_)F_3-*x*_ and 413 K for (NH_4_)_2_(Mg_1-*x*_Li_*x*_)F_4-*x*_ for 1—5 h.

K(Mg_0.9_Li_0.1_)F_2.9_ and K_2_(Mg_0.9_Li_0.1_)F_3.9_ were synthesized from KF (99%, Wako Pure Chemical Industries, Ltd., Japan), MgF_2_ (99.9% up, Kojundo Chemical Laboratory Co., LTD., Japan), and LiF (99.98%, Sigma-Aldrich Japan, Japan) by solid state reaction. Powders of reagents were mixed in a stoichiometric ratio, and milled in Ar atmosphere by a planetary ball milling (P-6, Fritsch Japan Co., Ltd., Japan) at 600 rpm for 12 h. The mixtures were sintered at 923 K for K(Mg_0.9_Li_0.1_)F_2.9_ and 873 K for K_2_(Mg_0.9_Li_0.1_)F_3.9_ for 10 h in Ar atmosphere.

The obtained samples were characterized by X-ray diffraction (XRD, D2 phaser, Bruker AXS, Germany), scanning electron microscopy observation (SEM, JSM-7800F, JEOL, Japan), and electron probe micro analyzer (EPMA, JXA-8530F, JEOL, Japan). The thermal stability of the obtained samples was evaluated by thermogravimetry (TG, Cahn D200, Thermo Fisher Scientific K. K., Japan).

^1^H NMR measurements were performed using NMR spectrometer (ECA300, JEOL, Japan) with a resonance frequency of 282.8 MHz at 298–373 K. The chemical shifts were calibrated by Si(CH_3_)_4_.

### Electrical conductivity measurements

The obtained NH_4_(Mg_1-*x*_Li_*x*_)F_3-*x*_ and (NH_4_)_2_(Mg_1-*x*_Li_*x*_)F_4-*x*_ powders were pelletized at 200 MPa by a cold isostatic pressing method. Au thin film electrodes were sputtered on the both sides of the dense pellets. Electrical conductivities were evaluated from AC electrochemical impedance spectroscopy (EIS) at 303–343 K in N_2_ gas with 30–50 mV of amplitude with frequency of 4.0 × 10^7^ to 1 Hz by using the impedance analyzer (Alpha-A, Novocontrol Technologies GmbH & Co. KG, Germany).

The powders of K(Mg_0.9_Li_0.1_)F_2.9_ and K_2_(Mg_0.9_Li_0.1_)F_3.9_ were pelletized at 200 MPa by a uniaxial pressure, and sintered at 1073 or 873 K for 10 h. Electrical conductivities of K(Mg_0.9_Li_0.1_)F_2.9_ and K_2_(Mg_0.9_Li_0.1_)F_3.9_ were evaluated from AC EIS at room temperature − 788 K in Ar atmosphere by using a potentiostat (VersaSTAT, Princeton Applied Research, USA).

To confirm the dominant fluoride ion conduction, DC polarization measurements were performed by using the blocking cell consisting of Pb/PbSnF_4_/sample/PbSnF_4_/Pb at room temperature − 423 K under vacuum. Schematic illustration of the blocking cell was given in Fig. S9. The current for DC polarization measurements was 10 or 20 mA.

## Supplementary Information

Supplementary Information.
