# Supplementary material for: Fast fluoride ion conduction of NH4(Mg1-xLix)F3-x and (NH4)2(Mg1-xLix)F4-x assisted by molecular cations
Source: Sci Rep. 2022 Apr 8;12:5955. doi: 10.1038/s41598-022-09835-0 (PMC8993874; doi:10.1038/s41598-022-09835-0)
Supplement: Supplementary file 1 — Supplementary Information. [file 41598_2022_9835_MOESM1_ESM.docx]

Supplementary information (SI)

Fast Fluoride Ion Conduction of NH_4_(Mg_1-_*_x_*Li*_x_*)F_3-_*_x_*

and (NH_4_)_2_(Mg_1-_*_x_*Li*_x_*)F_4-_*_x_* Assisted by Molecular Cations

Kota Motohashi^1, 2^*, Yosuke Matsukawa^1^, Takashi Nakamura^3^,

Yuta Kimura^3^, Naoaki Kuwata^4^, Yoshiharu Uchimoto^5^, and Koji Amezawa^3^*

^1^ Graduate School of Engineering, Tohoku University, 6-6-4 Aramaki Aza Aoba Aoba-ku, Sendai, Miyagi 980-8579, Japan.

^2^ Present address: Graduate School of Engineering, Osaka Prefecture University, 1-1 Gakuen-cho, Naka-ku, Sakai, Osaka 599-8531, Japan.

^3^ Institute of Multidisciplinary Research for Advanced Materials, Tohoku University,

2-1-1 Katahira Aoba-ku, Sendai, Miyagi 980-8577, Japan.

^4^ National Institute for Materials Science, 1-1 Namiki, Tsukuba, Ibaraki 305-0044, Japan.

^5^ Graduate School of Human and Environmental Studies, Kyoto University, Yoshida-nihonmatsu cho, Sakyo-ku, Kyoto 606-8501, Japan.

^*^ Corresponding author

Kota Motohashi

Graduate School of Engineering, Osaka Prefecture University, 1-1 Gakuen-cho, Naka-ku, Sakai, Osaka 599-8531, Japan.

Tel. +81-72-254-9333

Fax +81-72-254-9910

E-mail: kota.motohashi@chem.osakafu-u.ac.jp

Koji Amezawa

Institute of Multidisciplinary Research for Advanced Materials, Tohoku University,

2-1-1 Katahira Aoba-ku, Sendai, Miyagi 980-8577, Japan.

Tel. +81-22-217-5340

Fax +81-22-217-5343

E-mail: koji.amezawa.b3@tohoku.ac.jp

**The results of electromotive force measurements of the concentration cell**

In order to further confirm the dominant fluoride ion conduction, we measured electromotive force (*emf*) of the concentration cell composed of M_1_F*_x_*-M_1_/NH_4_(Mg_0.9_Li_0.1_)F_2.9_/M_2_F*_x_*_’_-M_2_. Mixtures of metal fluoride and metal, such as PbF_2_-Pb, FeF_3_-Fe and BiF_3_-Bi, were used as an electrode of the concentration cell. The *emf* of the concentration cell can be expressed by the difference between the fluorine activities in the electrodes, if the transference number of fluoride ion in the electrolyte in unity.

$$emf=-\frac{RT}{F}ln\left( \frac{a_{F_{2}, electrode 1}}{a_{F_{2}, electrode 2}} \right) (1)$$

where *R*, *T*, $a_{F_{2}, electrode 1}$, $a_{F_{2},electrode 2}$, are the gas constant, temperature, the equilibrium fluorine activity of the electrode 1, and that of the electrode 2, respectively. The equilibrium fluorine activity in the mixture of MF*_n_*/M (MF*_n_*: metal fluoride, M: metal) was calculated from the formation energy of MF*_n_* in thermodynamic database.^1^ *Emf* measurements were carried out under vacuum at 423 K. Figure S10 shows the results of the *emf* measurements. The purple and orange plots represent the results when iron-iron fluoride and lead-lead fluoride were used as one side of the electrodes, respectively. The other side of the electrodes was bismuth-bismuth fluoride in both measurements. The observed *emf* values were 440 and 280 mV, which were close to the theoretical ones, 483 and 322 mV, respectively. The transference numbers of fluoride ion conduction can be estimated to be approximately 0.91 from the iron-iron fluoride/bismuth-bismuth fluoride cell and 0.87 from lead-lead fluoride/bismuth-bismuth fluoride cell. These results suggested that NH_4_(Mg_0.9_Li_0.1_)F_2.9_ exhibited dominant fluoride ion conduction.

Fig. S1. SEM images and EPMA composition profiles of cross sectional of the compacts of NH_4_(Mg_0.8_Li_0.2_)F_2.8_.


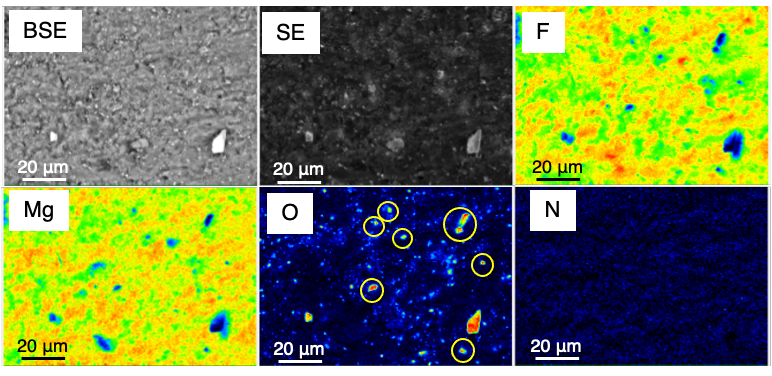


Fig. S2. SEM images of the fractured surfaces of the compacts of (a) NH_4_(Mg_0.8_Li_0.2_)F_2.8_ and (b) (NH_4_)_2_(Mg_0.85_Li_0.15_)F_3.85_.


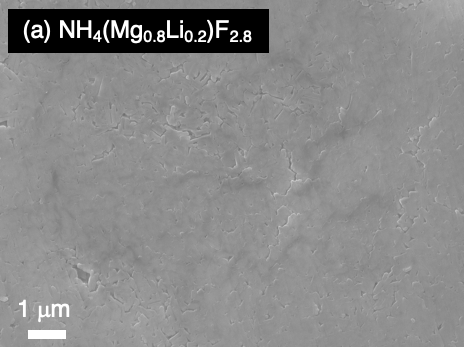

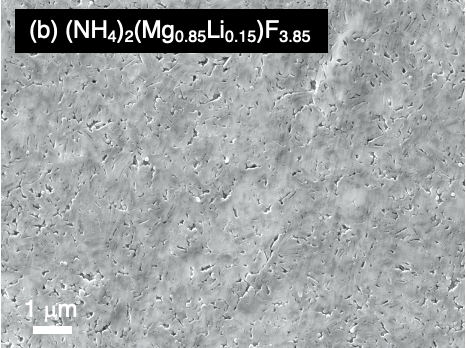


Fig. S3. Thermogravimetric curves of (a) NH_4_MgF_3_ and (b) (NH_4_)_2_(Mg_0.8_Li_0.2_)F_3.8_, and powder X-ray diffraction patterns of (c) NH_4_MgF_3_ and (d) (NH_4_)_2_(Mg_0.8_Li_0.2_)F_3.8_ before and after thermogravimetry measurements.


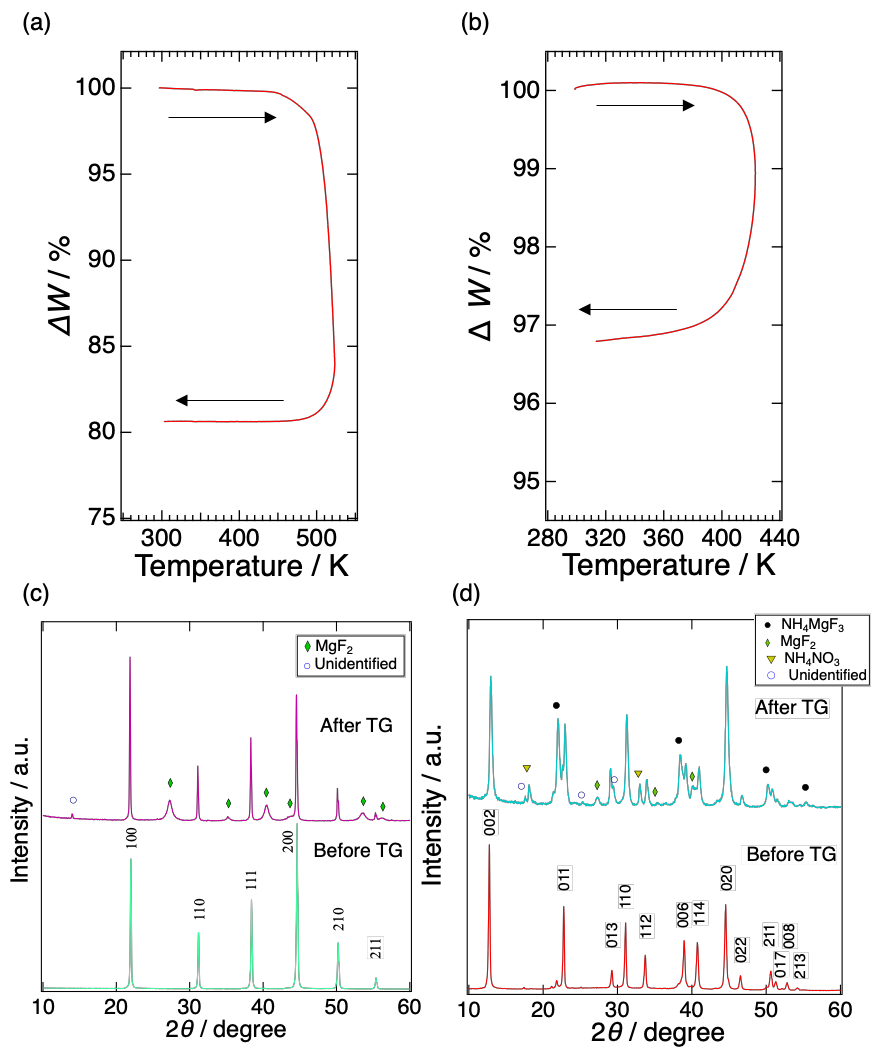


Fig. S4. (a) Nyquist plots obtained in AC EIS measurements at various temperatures, the voltage transient curves observed in DC polarization measurements at (b) 348, (c) 373, (d) 398, and (e) 423 K with a Pb/PbSnF_4_/NH_4_(Mg_0.9_Li_0.1_)F_2.9_/PbSnF_4_/Pb cell.


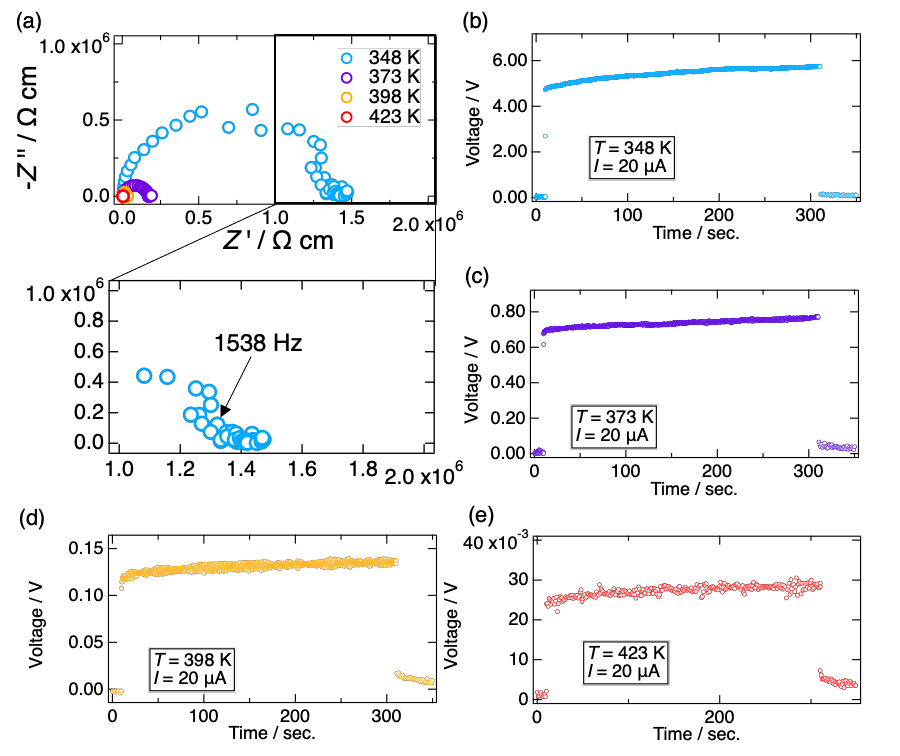


Fig. S5. (a) Nyquist plots obtained in AC EIS measurements at various temperatures, the voltage transient curves observed in DC polarization measurements at (a) 313, (b) 318, (c) 322, (d) 326, (e) 332, (f) 336, and (g) 341 K with a Pb/PbSnF_4_/(NH_4_)_2_(Mg_0.95_Li_0.05_)F_3.95_/PbSnF_4_/Pb cell.


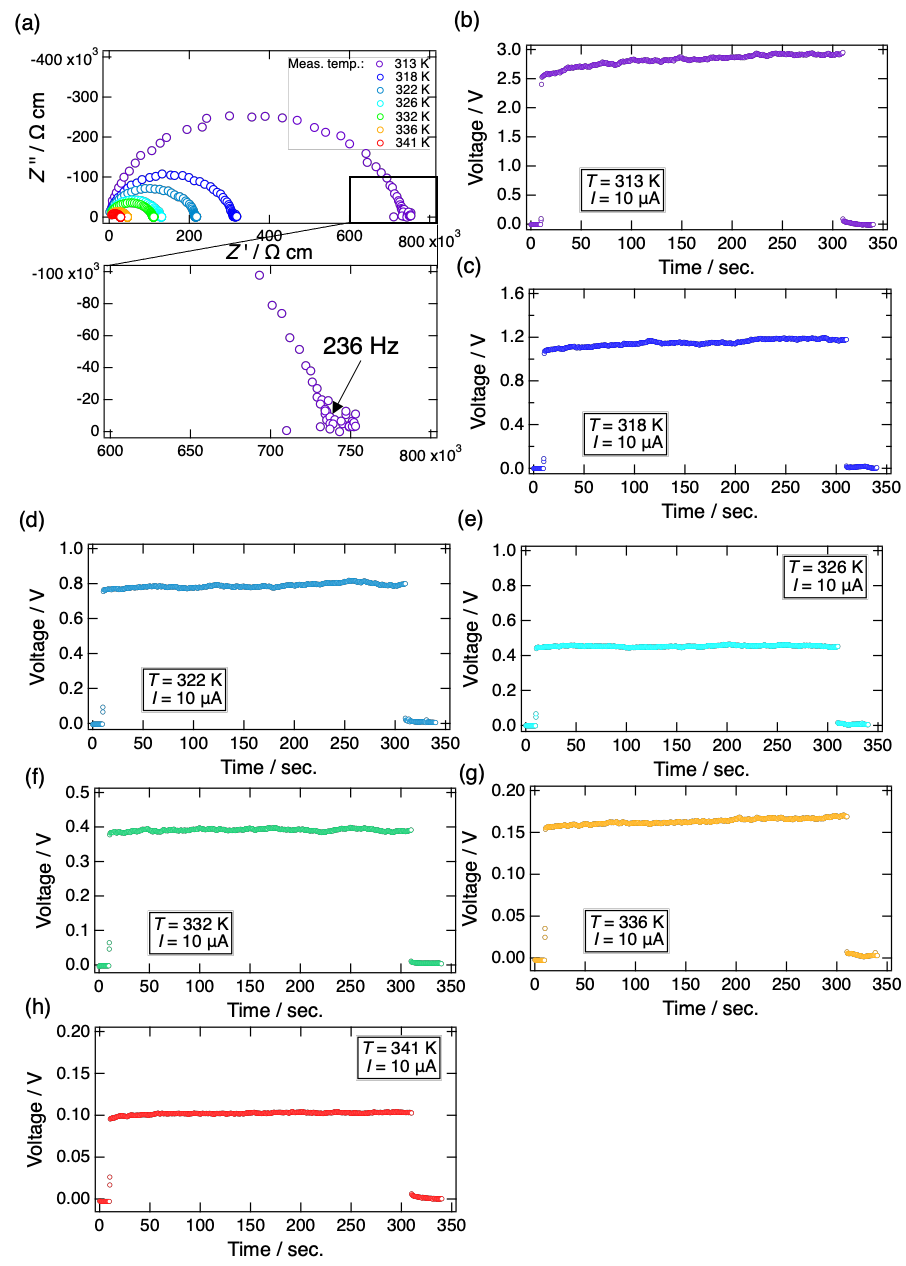


Fig. S6. Temperature dependence of electrical conductivities of (NH_4_)_2_(Mg_0.9_Sc_0.1_)F_4.1_.

Fig. S7. Temperature dependence of electrical conductivities of K(Mg_0.9_Li_0.1_)F_2.9_ and K_2_(Mg_0.9_Li_0.1_)F_3.9_.


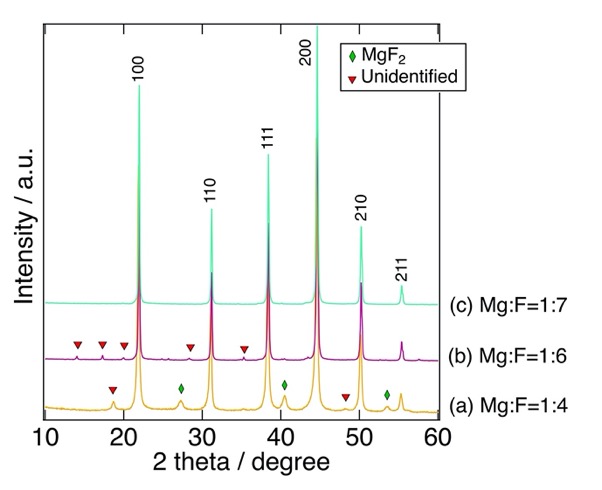


Fig. S8. Powder X-ray diffraction patterns of NH_4_MgF_3_ synthesized with different mixing molar ratio of NH_4_F.


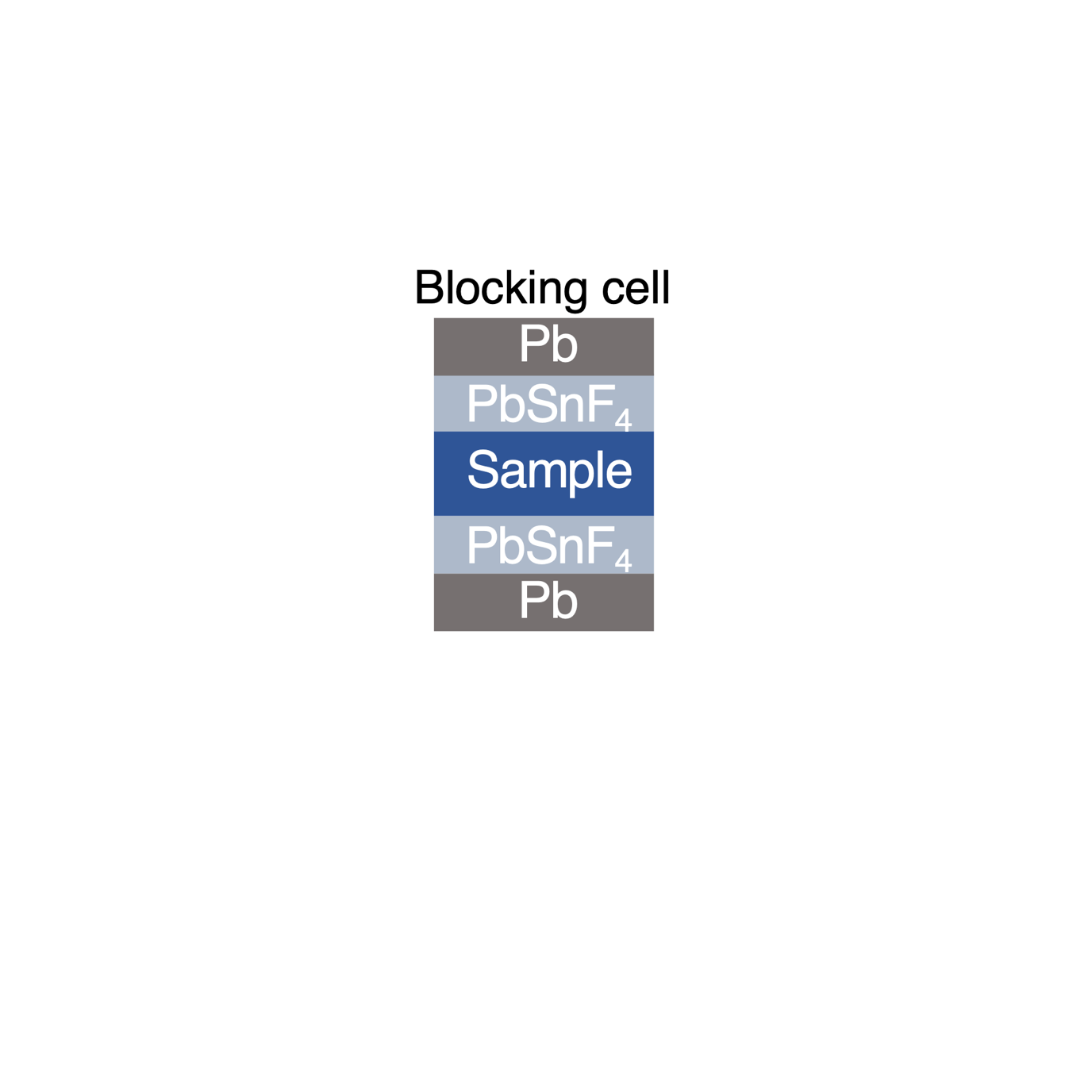


Fig. S9. Schematic illustration of the blocking cell.


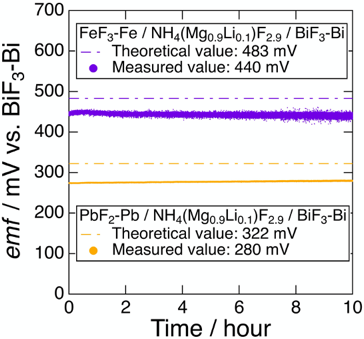


Fig. S10. Electromotive forces observed with the concentration cells of FeF_3_-Fe/NH_4_(Mg_0.9_Li_0.1_)F_2.9_/BiF_3_-Bi and PbF_2_-Pb/NH_4_(Mg_0.9_Li_0.1_)F_2.9_/BiF_3_-Bi at 423 K.

Fig. S11. Powder X-ray diffraction patterns of K(Mg_0.9_Li_0.1_)F_2.9_ and K_2_(Mg_0.9_Li_0.1_)F_3.9_ in this work.

| **Material** | ***E*_a_ / eV** |
| --- | --- |
| NH_4_(Mg_0.9_Li_0.1_)F_2.9_ | 1.09 |
| NH_4_(Mg_0.8_Li_0.2_)F_2.8_ | 0.93 |
| (NH_4_)_2_MgF_4_ | 0.84 |
| (NH_4_)_2_(Mg_0.95_Li_0.05_)F_3.95_ | 0.9 |
| (NH_4_)_2_(Mg_0.9_Li_0.1_)F_3.9_ | 0.96 |
| (NH_4_)_2_(Mg_0.85_Li_0.15_)F_3.85_ | 1.03 |
| (NH_4_)_2_(Mg_0.8_Li_0.2_)F_3.8_ | 0.98 |
| PbSnF_4_ | 0.44 |
| Ce_0.9_Sr_0.1_F_2.9_ | 0.47 |
| Ba_0.6_La_0.4_F_2.3_ | 0.59 |
| Ba_0.7_Sb_0.3_F_2.3_ | 0.65 |
| CeF_3_ | 0.50 |
| BaF_2_ | 0.64 |

Table S1. Activation energies of electrical conductivities in NH_4_(Mg_1-_*_x_*Li*_x_*)F_3-_*_x_*, (NH_4_)_2_(Mg_1-_*_x_*Li*_x_*)F_4-_*_x_*, and various fluoride ion conductors.

Reference in supplementary Information

[1] Thermodynamic database MALT group, Thermodynamic Database MALT for Windows, Kagaku Gijutsu-Sha, 2005 (CD-ROM), available from, <https://www.kagaku.com/malt/index.html>, Accessed data: 21 May 2019.
